# Playing for progress: policy advocacy in sport for development

**DOI:** 10.3389/fspor.2025.1546222

**Published:** 2025-03-13

**Authors:** Louis Moustakas, Sarah Carney, Sally-Ann J. Fischer, Alana Richardson, Karen Petry, Arnost Svoboda, Ansley Hofmann, Ben Sanders

**Affiliations:** ^1^ Institute of Sport and Sustainable Development, University of Applied Sciences Kufstein, Kufstein, Austria; ^2^ UNESCO Chair in Inclusive Physical Education, Sport, Fitness and Recreation, Munster Technological University, Tralee, Ireland; ^3^ Institute for European Sport Development and Leisure Studies, German Sport University, Cologne, Germany; ^4^ Department of Social Studies in Kinanthropology, Palacký University Olomouc, Olomouc, Czechia; ^5^ FairPlayPoint, Prague, Czechia; ^6^ The International Platform on Sport and Development, Copenhagen, Denmark

**Keywords:** social impact, power, ideology, funding, sustainable development, politics, advocacy

## Abstract

Sport for development (SFD) has emerged as a significant field of activity and has increasingly been critiqued for focusing on micro-level initiatives as a remedy to larger scale social or structural issues. This has led numerous scholars to propose more political meso or macro level approaches to deliver improved, sustainable outcomes. One such solution involves direct engagement in policy advocacy to support, and influence, policies that can directly benefit participants in SFD programmes. Against this background, our paper maps policy advocacy in the SFD field. Using results generated from a survey initiated in the context of a pan-European project, we map out the policy advocacy areas, activities and relationships within the sector. Our results show that most organisations engage in some form of advocacy, but much of this appears limited to the kind of self-interested advocacy that is designed to secure funding for organisational activities. Based on this, we argue that SFD actors should also engage in more progressive advocacy and suggest how research, as well as educational programming, can support this shift.

## 1 Introduction

Sport for development (SFD) has developed into a significant field of activity across the globe. With programmes and initiatives focusing on a wide range of thematic areas, the SFD movement concerns itself with intentionally using sport and physical activity to contribute to outcomes beyond the playing field, including to the Sustainable Development Goals (SDGs) (1). Though many individual programmes offer promising outcomes around the development of individual skills and greater awareness of social topics (2), the field has also faced numerous important criticisms. Broadly speaking, SFD has been critiqued for predominantly focusing on offering micro-level initiatives as a remedy to larger scale social or structural issues such as social cohesion, gender equality or poverty (3). As such, many authors have highlighted how programme results often fail to generate sustainable impact in their wider communities and, instead, risk reproducing existing, oft-detrimental neoliberal power structures (4, 5).

Likewise, scholars have proposed a range of solutions to address these concerns. By and large, these solutions highlight the need for politically informed programming, for instance through the use of critical pedagogy within programme delivery and further engagement of stakeholders at all levels of society (4, 6, 7). One particular proposal highlights the need for SFD organisations to move away from a focus on mere programme delivery and engage directly with decision makers through policy advocacy (8, 9). This suggestion goes beyond the *self-interested,* or sometimes even evangelical, advocacy connected to “making a case for sport”—a typical concern of a still young sector often facing precarious funding—and instead implies a *progressive* advocacy for policies that may benefit programme participants and communities at large (10, 11).

Despite calls for more political and advocacy engagement, SFD research has continued to primarily focus on the sporting or pedagogical aspects of its work, and how the SFD sector engages in this advocacy remains unclear. Yet, to effectively build upon these potential solutions, it is crucial to have an adequate understanding of the current status quo in the field. Only from this informed vantage point can advocacy be more effectively developed and deployed by actors operating at the intersection of sport and development.

With that in mind, the goal of the following paper is to map policy advocacy in the SFD field. Using results from a survey initiated in the context of a pan-European project, we map out the policy advocacy areas, activities and relationships within the sector. This project, entitled Policy Advocacy for Sport and Society (PASS), aims to raise awareness of policy advocacy within the SFD sector while also developing learning materials and usable tools to support the actual implementation of advocacy activities within the sector. In addition, beyond the more practical relevance of this work as described above, this research also responds to calls to more closely investigate the relationship between non-governmental and political actors in SFD (12, 13). As such, our ambitions here are primarily exploratory, as we seek to build an understanding of current advocacy practice and set the table for future work in this area.

Moving forward, our paper progresses in four steps. First, we will elaborate on the critiques levied against the SFD sector and situate more precisely our understanding of policy advocacy, as well as how it could represent a potential response to these criticisms. Second, we will present the overall background and methodology informing this paper. Third, we will present the descriptive results of our survey as well as a typology of common advocacy behaviours. Finally, we will critically discuss these results, reflecting on implications for research and the SFD sector as whole.

## 2 Policy advocacy and sport for development: mechanisms and importance

One of the main concerns about the current overall implementation of SFD revolves around the predominantly micro-level, individual focus of programming. At its most critical, such an approach is seen as concealing the role of privilege, institutions and social structures in reproducing inequalities, and instead shifts responsibility, and by extension blame, entirely onto individual participants (4, 14). At a more pragmatic level, there is also a sense that this lack of engagement with policy limits the potential to develop programming that is aligned with current policy realities and advocates for policies that benefit programme participants, thus restricting the sustainability of programme outcomes. For instance, Moustakas et al. (9) highlight how, in the context of a sport for employability programme, immigrant participants faced often confusing, contradictory requirements concerning the obtention of a work permit, which eventually served to push them back into informal, lower-paid work. Though one individual programme may not be able to reform immigration processes overnight, this presents an example of the kind of policy issues where SFD organisations could act on behalf of their participants. Other authors likewise highlight the need for programmes to take into account existing policies and work within political structures in order to ensure the sustainability of their outcomes (6, 7).

Policy advocacy, broadly defined as the active support of specific policies or a group of policies, represents a potential pathway for this kind of policy engagement. Policy advocacy activities are characterised as public or citizen-led and often occur within the sphere of non-governmental organisations (NGOs) (15–17). As such, policy advocacy is often presented as a bottom-up process that contrasts with the top-down nature of governments (16). The potential benefits of advocacy are manifold, though there are also numerous inherent risks and challenges. At a conceptual level, policy advocacy can be an important vehicle to recalibrate power relations in favour of communities or programme participants. Programme participants within SFD organisations, or social/human service organisations more generally, often come from marginalised groups that face often challenging structural conditions and frequently have little say in formal decision-making processes [see, e.g., (18)]. Policy advocacy, especially the kind of progressive policy advocacy that actively works with and for participants, can challenge these power relations by actively engaging with, or against, policymaking processes and, ultimately, support systemic change (15). In other words, policy advocacy can represent a variety of strategies to articulate, and reclaim, power through or over ideas and reframe what is considered common knowledge or common sense amongst the public and policy makers (19). At a more pragmatic level, advocacy can help identify shared interests between parties, support consensus building, enhance organisational and institutional credibility and foster improved policymaking processes [(20–22); for a conceptual review, see (17)]. As Mosley (23) summarises, policy advocacy has the potential to “both strategically position organizations in their environment and promote client well-being” (p. 57). Along these lines, in (community) sport, advocacy has been increasingly positioned as an important vehicle to “make the case for sport” while also securing wider community benefits (11, 24). Nonetheless, organisations in the social sector, like many of those operating in sport or the SFD field, face numerous challenges implementing policy advocacy activities. Organisations often operate with limited, irregular budgets, which restricts their ability to maintain consistent advocacy efforts (25). Advocacy often involves challenging powerful stakeholders, such as corporations or government bodies, whose interests may conflict with an organisation's goals and therefore lead to opposition or competition (21). Identifying and accessing these stakeholders can prove challenging in the first place, especially when organisations operate outside of existing political networks. In other circumstances, it may be hard to challenge certain stakeholders as those same stakeholders may be directly involved in the funding of SFD programming, reducing organisations' perceived range of advocacy opportunities.

To better understand and conceptualize how advocacy occurs, the framework proposed by Gen and Wright (17) offers a valuable starting point. The authors synthesize insights from both practitioner and academic literature to present a comprehensive model of policy advocacy, outlining its inputs, activities, outcomes, and impacts. They highlight the key inputs or competencies that legitimize advocacy efforts, the activities driven by these inputs, and the eventual outcomes and impacts. The framework is informed by multidisciplinary theories such as social capital and empowerment theory, which help explain the connections between these elements.

Specifically, inputs include resources, knowledge, relationships, and a sense of agency. Activities range from engaging decision-makers and the public to information campaigns, policy monitoring, defensive actions, and reform efforts. Outcomes are divided into two categories: proximal (short-term) outcomes, such as improving the democratic environment or shifting stakeholder perspectives, and distal (long-term) outcomes, like policy adoption or implementation. Impact refers to direct changes for the target populations, as well as shifts in related services and systems.

A simplified version of the framework, with brief descriptions of each element, is provided in Table 1. Overall, this framework outlines the diverse pathways advocacy can follow and demonstrates how various results—from raising awareness to achieving policy change—can emerge from advocacy efforts. Importantly, as explained in the following section, this framework informed the design of our mapping survey, particularly in relation to the policy advocacy activities of SFD organizations.

**Table 1:** Simplified policy advocacy framework, adapted from Gen and wright (17). Short summary descriptions are presented in the parentheses.

Inputs	Activities	Proximal Outcomes	Distal Outcomes	Impact
Material Resources (Tangible financial and physical resources)	Engaging the public (Awareness-raising and mobilisation of citizens)	Democratic environment	Policy adoption (Desired policy is conceived and adopted)	Public centred policymaking (Public and target population are actively involved in policy making)
Knowledge and Skills (Competencies and skills at the individual and group levels)	Coalition Building (Exchange of information and relationship-building with like-minded organisations)	Change in public views	Implementation change (Adopted policy leads to changes in practical implementation)	Changes for the target population (Conditions change for population targeted by policy)
People and Relationships (Strong, trusting relationships)	Engaging decision makers (Engaging and building rapport with relevant decision-makers)	Change in decision-maker views		Changes in services and systems (Conditions change services or systems targeted by policy)
Sense of agency (a belief that actions may have an impact on policy)	Information campaigning (Research and rhetoric to persuade and support policy advocacy)			
	Reform efforts (Attempts to change policy through pilots, demonstrations or litigation)			
	Defensive activities (engaging opposing factions in discourse)			
	Policy monitoring (Monitor policy implementation to support learning or apply pressure)			

**Table 2:** Overview of variables used in typology formation.

Policy advocacy focus	• Sport policy • Other policy areas • Funding
Policy advocacy activities	• Awareness raising campaigns targeting the general public. • Networking with policy makers. • Meetings to share information with policy makers. • Research on own programmes to develop evidence of benefits. • Meetings to discuss problems and look for solutions with policy makers. • Building coalitions of like-minded groups. • Awareness raising campaigns targeting decision makers. • Engaging your members the public to take other actions. • Taking part in consultations or debates concerning policy development. • Research on situation of programme participants to identify (policy) needs. • Sharing briefing documents with policy makers. • Initiating pilot or demonstration projects to showcase alternative (policy) approaches. • Monitoring implementation of relevant policies. • Engaging your members the public to write letters, sign a petition or take part in a demonstration. • Taking part in legal action or litigation to achieve policy changes.
Relationships with political levels	• Municipal level • Regional level • National level • National sport federations • International sport federations

## 3 Methodology

### 3.1 Background

This paper emerges from the context of our pan-European project called Policy Advocacy for Sport and Society [PASS; see (26)]. The project, which lasts three years between 2024 and 2026, features a consortium of three universities, two SFD NGOs, one transnational advocacy network, and one intergovernmental organisation. These partners are based across Europe, in Austria, Czechia, Denmark, Germany, Hungary, Ireland, and Switzerland.

This project aims to raise awareness of policy advocacy within the SFD sector while also developing learning materials and usable tools to support advocacy activities within the sector. Concretely, this project will help identify good practice in the field and develop online learning materials to support organisations in the field. To ensure the relevance and appropriateness of these tools, an initial mapping survey was conducted to explore the current activities, goals and challenges associated with policy advocacy in the SFD sector. Initial results from this survey were presented in an applied project report and have been further analysed and developed for this paper [see (27)].

### 3.2 Data collection

An online structured survey was chosen as the most effective method to reach the SFD organisations targeted by our work. A 22-question survey was designed, with four separate sections: section one gathered basic organisation demographics, section two focused on current practice in relation to policy work, section three on the challenges for SFD actors to engage in policy advocacy, and finally section four aimed to identify training needs for the sector to more effectively engage in policy advocacy. Broadly speaking, sections one and two form the basis for the following paper and, except for an open-ended question at the end, all questions are multiple choice or Likert-scale questions. Section two surveys the levels at which SFD organisations engage with policymakers (e.g., municipal governments, national governments, sport federations), the quality of their relationships at those different levels, the kind of policy advocacy activities deployed, and the general goals of their advocacy. Here, it is worth noting that we explicitly included national and international sport federations as a type of policymaking organisation that may be on the receiving end of advocacy activities. Sport federations have a high degree of autonomy, set important rules and policies within sport, and Olympic committees or sport confederations represent a sort of de-facto sport ministry in many contexts [see e.g., (28)].

A predominantly convenience and purposive sampling approach was taken. Our survey aimed to capture SFD stakeholders engaging in advocacy at different levels (e.g., national, international) and within different organisational types [cf. (29)], thus giving us a broad based view of policy advocacy within the field. The survey was shared within the immediate networks of the project consortium members, as well as via thematic networks relevant to the field. This included via the e-mail newsletter of SportandDev.org, as well as in direct e-mails to members of CommonGoal, the Council of Europe's Sport Integration Platform, and organisations associated with the Sport Diplomacy academy. CommonGoal is a transnational advocacy and funding organisation uniting organisations using football within their SFD activities, whereas the Sport Integration Platform and Sport Diplomacy Academy both contain databases of mainly implementing organisations. Targeting this mix of organisations allowed us to reach a range of implementers, foundations and networks, mirroring the diverse, disparate nature of the SFD field. In addition, to further maximise uptake among partners' networks, the survey was translated and made available in English, French, German, Czech and Hungarian. All participants provided informed consent directly within the survey form, and the research received ethical approval from the German Sport University (222/2023).

### 3.3 Data analysis

Data were extracted and initially organised into an Excel table, and later also converted into formats suitable for analysis within SPSS and MaxQDA. The data analysis followed two broad steps. First, descriptive statistics were generated summarising the key top-line findings related to each question.

Second, to understand typical patterns within the sample, a typology was developed to identify constellations of policy advocacy behaviours within the group of respondents. To do so, we followed Kluge's (30) four steps of empirically grounded type construction and complemented these with additional statistical and group checks. These steps include defining the properties and dimensions that form the basis for the typology, grouping the cases according to those properties, analysing the relationship and constructing types.

For the first step, we decided to restrict our focus on the policy advocacy activities, type of activities used and relationships with existing policy stakeholders. These variables, which we summarise in Table 2, were selected as all respondents provided standardised responses in these areas that simplified analysis while still allowing us to assess how certain activities influenced different relationships. Second, we used various techniques to group our sample according to these properties, including statistical tests such as K-Means or Two-Step Clustering, as well as by generating visual clusters of the organisations or codes in MaxQDA using simple matching. These different tests were used as, for the most part, they are able to accommodate the binary nature of the data considered for the typology (31, 32). This step allowed us to create a variety of typologies for comparison and develop initial descriptions of those typologies. In a further step, we discussed the construction of typologies and refined their descriptions during a project meeting in October 2024. Ultimately, we settled on a final typology of four advocacy types that we describe further in the results below.

## 4 Results

### 4.1 Organisational background and engagement

In total, 115 full responses were received. Most organisations represented are development organisations (60%), with slightly more engaging at local level (23%) than at national (21%) or international levels (16%). In terms of geographic distribution, a total of 27 unique countries could be identified, with a plurality of responses (circa 48%) coming from Europe, which is likely a reflection of the European nature of the project and its funding. Germany (16 responses), Hungary (7), the Czech Republic, Kenya, and Uganda (5 responses each) were the most well-represented countries. As for the organisational forms represented, a plurality of responses came from local non-governmental or sport (for development) organisations (48), followed by national-level non-governmental or sport (for development) organisations (30). Responses also came from international NGOs (4) or SFD (18) organisations, educational organisations and even two for-profit consultancies involved in SFD.

**Figure 1:**
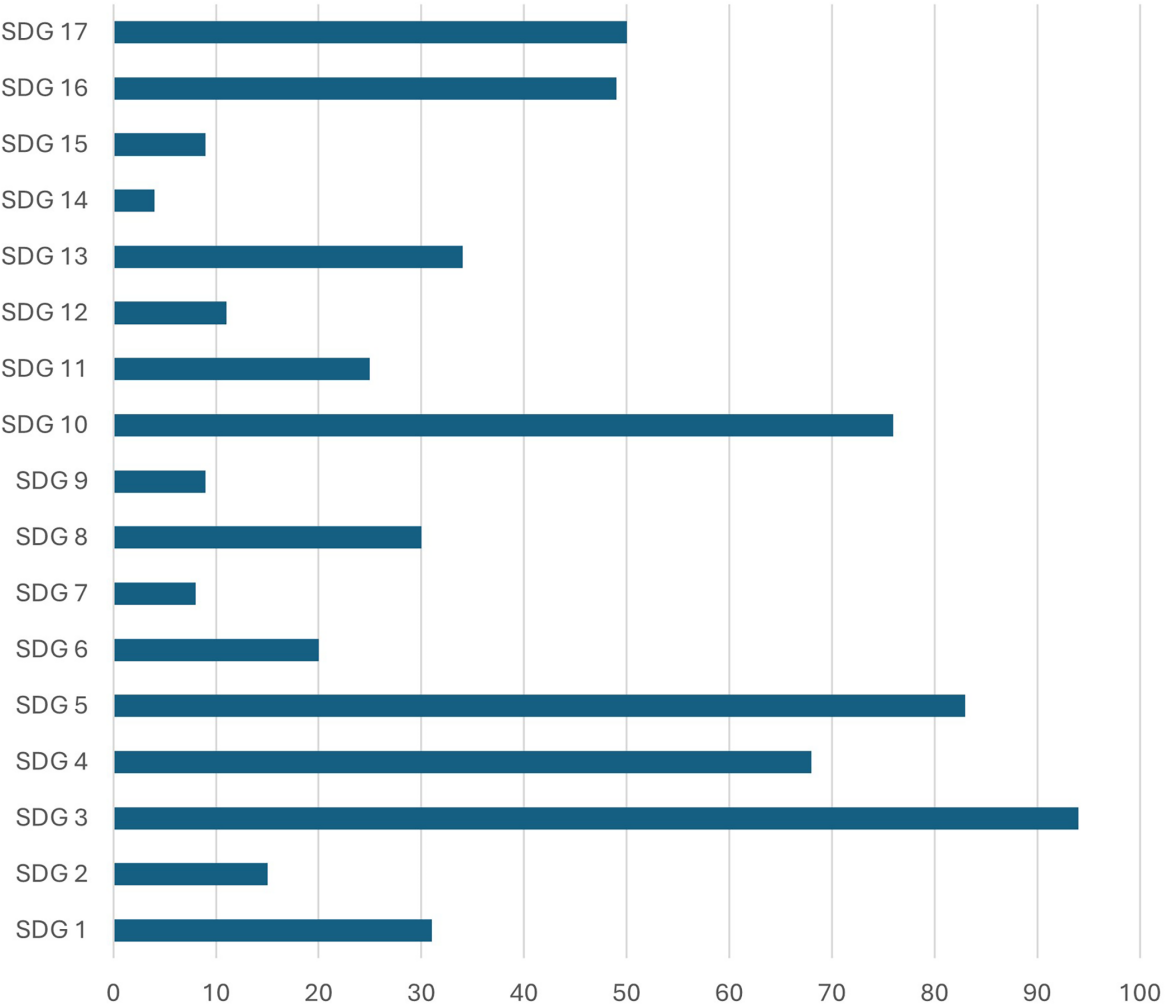
Focus on different sustainable development goals (SDG) within the work of responding organisations. Multiple answers per respondent were possible.

Regarding the types of participants programmes target, children in general were the most common group (82 responses), followed by women and girls (78) and socio-economically disadvantaged groups (66). The full range of beneficiaries targeted are displayed in Table 3. Finally, in terms of the sustainable development goals (SDGs) associated with the programmes, a plurality of organisations focusses on good health and wellbeing (94), gender equality (84), and reduced inequalities (76). The full range of SDGs targeted by organisations are presented in Figure 1.

**Table 3:** Main beneficiary target groups of activities delivered or supported by responding organisations. Multiple answers per respondent were possible.

Target group	#
Children	82
Women and girls	78
Socio-economically disadvantaged groups	66
People with physical disabilities	59
People with intellectual disabilities	34
People forced to flee (asylum seekers, refugees, etc.)	32
Ethnic minorities	29
LGBTI + community	11
Roma and or Travellers	9

### 4.2 Policy advocacy engagement

Overall, two-thirds of our sample (66%) report engaging in policy advocacy, with another 18% claiming they do not engage at all and 16% reporting not being sure. In terms of policy advocacy focus, organisations focus largely on non-sport policy areas (87), followed by funding (69) and sports policy (54).

**Figure 2:**
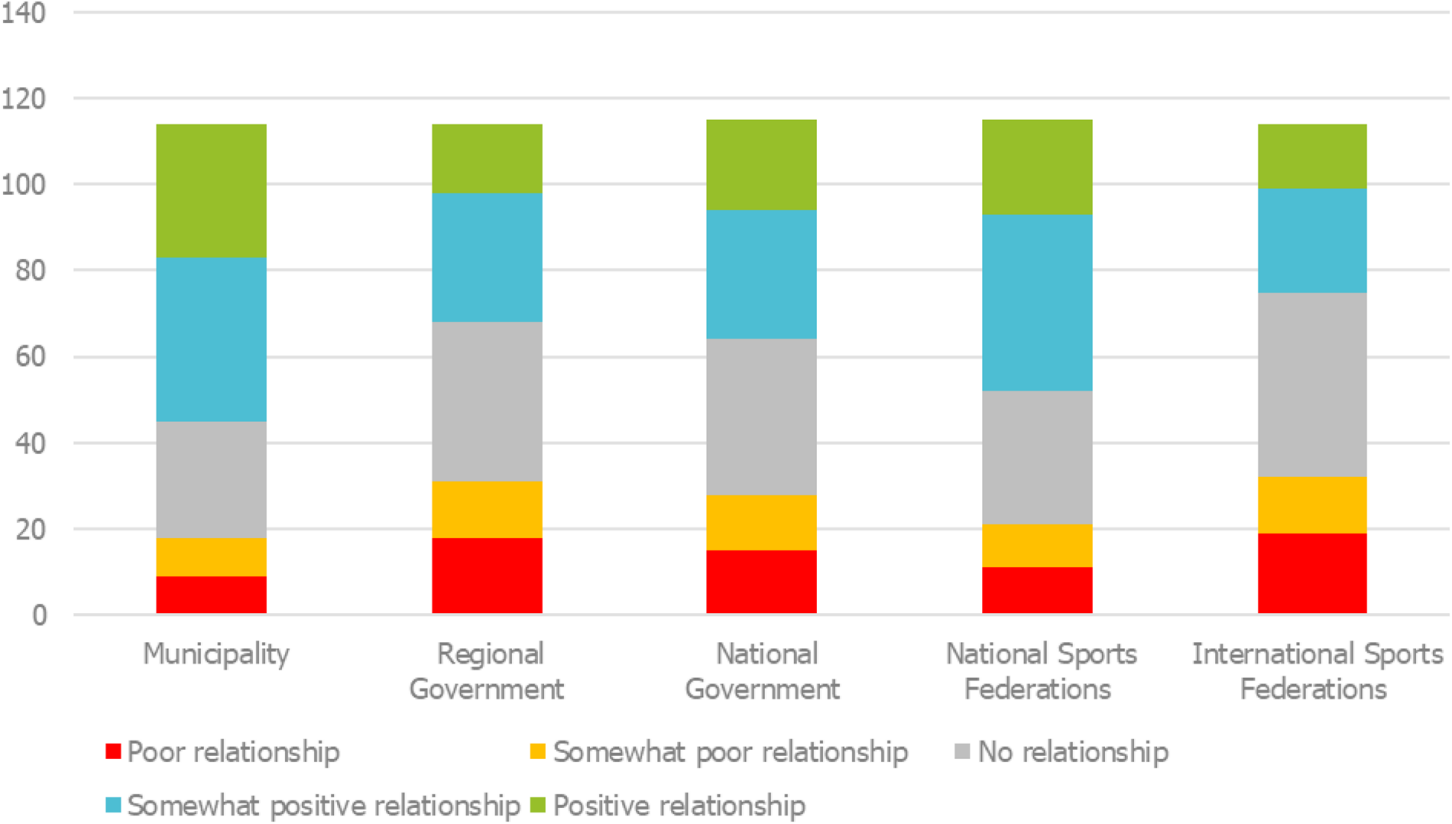
Presence and quality of respondent's relationships at different political levels.

This advocacy predominantly occurs at the municipal level, where most respondents (69) report having positive or somewhat positive relationships with policymakers. In contrast, international sport federations and national governments remain more distant, with majorities reporting either no or poor relationships. The quantity and quality of relationships with the different political levels are depicted in Figure 2.

In terms of activities, which we present in Table 4, public awareness-raising as well as various other infomation sharing and networking activities represent the most typical activity types. More direct or confrontational tactics, such as demonstrations, policy monitoring or legal action remain comparatively infrequent.

**Table 4:** Policy advocacy activities of survey respondents developed based on Gen and wright (17). Multiple answers per respondent were possible.

Action	Total
Awareness raising campaigns targeting the general public.	81
Networking with policy makers.	70
Meetings to share information with policy makers.	66
Research on own programmes to develop evidence of benefits.	64
Meetings to discuss problems and look for solutions with policy makers.	63
Building coalitions of like-minded groups.	62
Awareness raising campaigns targeting decision makers.	58
Engaging your members the public to take other actions.	50
Taking part in consultations or debates concerning policy development.	47
Research on situation of programme participants to identify (policy) needs.	45
Sharing briefing documents with policy makers.	39
Initiating pilot or demonstration projects to showcase alternative (policy) approaches.	37
Monitoring implementation of relevant policies.	32
Engaging your members the public to write letters, sign a petition or take part in a demonstration.	29
Taking part in legal action or litigation to achieve policy changes.	17
Others	3

Finally, in terms of barriers to advocacy work, organisations predominantly indicated that insufficient resources (82) were a significant challenge, followed by sport not being taken seriously by policy actors (58) and a lack of expertise within the organisation (52). Interestingly, around 20% of organisations also indicated that advocacy is simply not a priority for them (25) or that they do not see advocacy as necessary or effective (21).

### 4.3 Policy advocacy typology

In order to expand our analysis beyond mere description, we further explored the data to identify potential common patterns of policy advocacy engagement and activities. Through our analysis described previously, we managed to develop four general types of advocacy behaviour within the responding sport for development organisation. The types are summarised in Table 5 and further tables depict the results from one of our statistical tests, the K-Means analysis, highlighting the percentage of organisations engaging in certain policy fields or activities is provided in the supplemental materials. Below, we describe and interpret these four advocacy types, situating the results of the analysis within broader literature on the topic.

**Table 5:** K-Means analysis describing, as a percentage of organisations, the focus, actions and relationship quality of organisations within each type. For this test, relationship quality was transformed into a binary variable (1–3 = 0, 4–5 = 1).

	Cluster	1: Moderate (24)	2: Disengaged (33)	3: Fully (16)	4: Challenged (34)
Focus	Sport policy	0.50	0.39	0.81	0.76
	Other policy areas	0.71	0.63	0.88	0.88
	Funding	0.67	0.32	0.63	0.82
Actions	Encouraging public action (demonstrations, letters)	0.04	0.20	0.13	0.52
	Encouraging public action (other)	0.42	0.34	0.31	0.61
	Coalition building	0.50	0.39	0.50	0.76
	Networking with policymakers	0.58	0.22	1.00	0.94
	Sharing information with policymakers	0.54	0.17	0.81	0.97
	Discussing solutions with policymakers	0.54	0.15	0.88	0.88
	Research on programme evidence	0.67	0.27	0.44	0.88
	Research on participant needs	0.50	0.17	0.25	0.64
	Briefing documents	0.25	0.10	0.25	0.73
	Awareness raising (policymakers)	0.17	0.27	0.75	0.91
	Awareness raising (public)	0.79	0.44	0.88	0.91
	Legal actions	0.08	0.07	0.19	0.27
	Pilot actions	0.08	0.07	0.56	0.67
	Consultations	0.17	0.17	0.75	0.70
	Monitoring	0.04	0.10	0.50	0.52
Relationship quality	Municipal gov.	0.75	0.44	1.00	0.52
	Regional gov.	0.83	0.17	0.81	0.18
	National gov.	0.96	0.17	0.69	0.30
	National sport federations	0.88	0.46	0.75	0.33
	International sport federations	0.83	0.22	0.13	0.24

#### 4.3.1 Fully engaged

These organizations demonstrate comprehensive policy engagement, maintaining regular involvement across all policy areas with particularly strong relationships at municipal and regional government levels. A distinctive characteristic is their notably limited or poor relationships with international sporting federations. Their advocacy approach primarily centres on direct networking and meetings with policymakers, complemented by extensive awareness-raising activities targeting both decision-makers and the general public. While some organizations in this category undertake evidence-building initiatives, policy monitoring, and pilot programs, these activities play a secondary role in their advocacy strategy. Notably absent from their approach are more confrontational methods. Demonstrations, protests, and legal actions are rarely employed, suggesting a preference for collaborative engagement or, alternatively, a strategy maintaining the position of an established organisation that understands and plays “by the rules”.

Advocacy work within this group likely serves the dual purpose identified in previous work, namely advancing self-interested objectives to secure organizational positioning and funding, while simultaneously pursuing broader progressive policy goals. The group's membership spans a diverse range of entities, including national and international Sport for Development organizations, transnational networks, educational institutions, and one intergovernmental body, making broad generalizations challenging. However, we can likely make one assumption about this group, namely that these organizations possess substantial resources and operational capacity, enabling them to maintain comprehensive advocacy efforts. As other research indicates, such a resource base appears to be a crucial enabling factor for their extensive policy engagement activities (23).

#### 4.3.2 Moderately engaged

These organizations maintain active engagement across general policy areas and funding, cultivating strong relationships at multiple governmental levels. However, their activity in the sport policy sphere is more limited. Their advocacy approach is distinguished by a primary focus on evidence-building research and public awareness campaigns, while direct policy engagement through networking and meetings with policymakers plays a rather limited role. As with the fully engaged group, there is minimal usage of more potentially confrontational tactics like protests or legal action.

The pattern of this group's activities suggests a predominantly self-interested form of advocacy, focused on promoting sport's perceived value and securing stable funding streams. While this approach is common among most Sport for Development organizations, particularly given the sector's characteristically unstable and short-term funding landscape [see e.g., (34)], these organizations appear to make it their primary advocacy focus. The strategy here appears to revolve around developing evidence, communicating findings to policymakers, and working specifically with non-sport policy actors to demonstrate sport's broader societal impact. Though we cannot definitively identify the content of this advocacy, this relatively limited engagement in other activities may reflect these organizations' resource constraints, suggesting that more progressive policy advocacy efforts take a back seat to activities focused on organizational survival and operational sustainability. This prioritization likely stems from practical necessity rather than strategic choice, with advocacy resources necessarily directed toward securing the organization's continued existence as opposed to focusing on longer-term, strategic or political objectives [cf. (35)].

#### 4.3.3 Disengaged

These organizations display minimal engagement across policy areas and governmental levels, with no clear pattern of consistent advocacy activities emerging from their work. The only notable commonality is public awareness raising, which appears in just under half of the organizations within this group. Their relationship-building efforts are similarly limited, showing meaningful connections only at the municipal level and with national sport federations, and even these are modest.

While our overall data suggests that various forms of advocacy are perhaps more widespread than initially assumed within the Sport for Development sector, this group reveals a significant subset of organizations whose advocacy engagement remains absent or superficial. The diversity of organizations in this group—spanning local, national, and international entities across Europe, sub-Saharan Africa, and other regions—makes it difficult to attribute this limited engagement to any one organizational form or geographical context. Two potential explanations develop for this pattern: first, these organizations may lack the financial and human resources widely recognized as prerequisites for effective policy advocacy work. Alternatively, some organizations may make a deliberate choice to minimize advocacy efforts, as hinted by our survey where nearly 20% of respondents indicated they do not view policy advocacy as a worthwhile investment of their resources.

#### 4.3.4 Challenged(ing)

These organizations engage broadly across policy domains but notably struggle to maintain positive relationships with policymakers—if that is even their goal in the first place—with only about half achieving even modest positive connections at the municipal level. Their advocacy strategy mirrors many aspects of fully engaged organizations, employing networking and information sharing with policymakers while also conducting participant-focused research and various political activities. Distinctively, this group shows the highest propensity for direct or confrontational advocacy methods, including demonstrations, protests, policy monitoring, and legal actions, though these remain minority approaches within the group.

Their consistently poor relationships with policymaking bodies may be a direct consequence of this more challenging advocacy style, potentially indicating a deliberate strategy of challenging the status quo. This approach aligns with pressure-based advocacy theories, where organizations combine indirect and direct methods to build momentum for policy change through multiple channels (17, 21). In practice, however, this can also bring significant challenges, such as the risk of losing funding opportunities by being too critical of potential funders or by engaging in coalitions with other, more explicitly critical advocacy organisations.

An alternative interpretation suggests these organizations may simply be struggling with advocacy effectiveness. Despite apparently dedicating significant resources and value to advocacy work, they may face fundamental challenges in identifying appropriate target groups, securing adequate resources, or tailoring their approaches effectively to their audience (23, 25). This raises an important possibility that, while these organizations clearly prioritize policy advocacy in their work, they may lack the critical mass of resources or expertise necessary to translate their high activity levels into effective policy influence and positive stakeholder relationships.

## 5 Discussion

In the above, we have attempted to map out the initial status of policy advocacy within SFD as well as cluster common patterns of activities to document potential types of policy advocacy for organisations in the field. In doing so, we have established a first baseline for the field while also exposing some of the inherent challenges or tensions related to this area. Before delving into this further, however, we are mindful that our work has some limitations that do restrict our ability to fully transfer these results to the field. Our primarily purposive and convenience sampling approach poses the most obvious limitations, leading to certain locations or organisation types being over, or under, represented. For instance, it is quite possible that international SFD organisations are overrepresented (*n* = 18) relative to local organisations (*n* = 48), especially considering the crucial role played by local organisations in both programme implementation and advocacy. Likewise, our sample did not capture a great number of organisations outside of Europe and sub-Saharan Africa, thus potentially restricting the applicability of our results outside of those areas. Translation of such a survey into other languages (e.g., Arabic, Spanish, Portuguese) as well as engagement with thematic networks from those regions, would certainly be of benefit for future work. In our attempt to keep the survey short and accessible, we did not assess organisational resources or the organisation's perceived skill/knowledge in advocacy. Yet, looking at the typology, these could prove to be important explanatory factors that drive organisations to exhibit the patterns inherent to any one group. Future work looking at policy advocacy in SFD would do well to be more mindful of these aspects, especially since literature regularly points to their importance in the advocacy context.

Overall, our work paints a mixed picture of advocacy in SFD. Though other authors have called upon the field to be more active in the policy sphere (8, 9), our data shows a perhaps higher than expected level of engagement, which is consistent with other authors' remarks on the active role of new social movements and campaign groups (36). After all, two-thirds of our sample reported being engaged in advocacy, and our typology presents various constellations of activities and relationships. It appears likely that a significant portion of this advocacy focuses on securing funding—an area 60% of organisations in our dataset already identify as a focus area—and communicating the value of the organisation's activities. In other words, looking at the predominant activities which focus largely on awareness-raising, networking and evidence-building, organisations mostly engage in self-interested advocacy, and less so in progressive advocacy. In a way, this makes some level of intuitive sense, as the precarity of funding and struggles for organisational survival are well documented within SFD (34, 37) and for social organisations more generally (35). Further, the resource intensive nature of progressive advocacy efforts may be difficult to pursue without initially securing funding. Additionally, as our typology shows, some organisations are only involved at a surface-level in advocacy and generally deploy very limited advocacy activities. Though political engagement and advocacy, in some ways, may be higher than expected, the core recommendation for SFD organisations to be more politically active remains valid even considering our results. Even organisations that do delve into progressive advocacy may sometimes do so to simultaneously ensure the sustainability of their programmes—as, for example, Right to Play reported doing to integrate its curriculum at the national level in other countries (38, 39).

From this, the logical next question is how SFD organisations can be better equipped and supported in delivering progressive policy advocacy with and for their participants. Certainly, our survey identifies some clear needs in terms of resources and knowledge related to policy advocacy, as well as a desire for policymakers to take sport organisations more seriously. Translating these needs into potential action, one obvious route would be the development of advocacy-specific funding streams that allow organisations to develop policy advocacy activities. At a minimum, as is our ambition with the PASS project, materials, tools and templates could be developed specifically for the SFD sector to reduce the threshold for policy advocacy engagement. Similarly, tailored capacity-building is needed for the sector, as many organisations here report lacking not only knowledge, but also not always seeing the value of policy advocacy. Yet, as noted above, policy advocacy can play a key role in delivering sustainable outcomes for participants and maintaining an organisation's position (17, 23). Future educational tools should not only develop key communication, assessment and planning skills, but also make a clear case for its value and situate advocacy within the broader policy context of SFD (40). Finally, increased coalition building in SFD would likely strengthen the sector's credibility and presence within policymaking arenas. Our results show that only about half of organisations build coalitions within SFD or beyond, indicating that there is still room for further coalition building in the sector. Although we note the presence of national SFD thematic networks in Germany and elsewhere that aim to more actively advocate for the sector (41), many actors are still mainly concerned with mapping and communicating the value of SFD within their local or national contexts. This perhaps suggests that there is a need for coalitions uniting implementing organisations in SFD and beyond that have a common thematic focus such as gender equality, human rights, or employability, allowing these organisations to unite their forces to advocate around more specific policy areas. Indeed, coalitions that integrate different actors across different levels may provide advantages whereby members can contribute varied, complementary resources, expertise, and networks (33).

Researchers need to play an important role in further documenting and developing policy advocacy within SFD. This engagement is all the more important when the evidence suggests that SFD actors operating on the grassroots level often lack the capacity to actively and effectively communicate with policymakers or engage in networks (42). In-depth exploration of current practices and successes are needed, along with insights from policymaker experiences with advocacy, to provide a clear picture of what works and does not within SFD-related policy advocacy. A key question here also concerns around which areas SFD are best suited to advocate for. Though not explicitly concerned with advocacy, previous research has drawn connections between SFD and advocacy in areas such as health (7) or employability (9). Further research would do well to explore where SFD-related advocacy efforts could have the greatest impact. Likewise, researchers could more closely explore the conditions and dynamics of coalition building within SFD. For instance, SFD actors may be active and responsive within their communities but not always well equipped for advocacy at the regional or national levels, and these actors may therefore benefit from collaboration with larger or more specialised advocacy organisations in the vein of Amnesty International, Terre des Hommes or others. How these coalitions or networks navigate their own internal power dynamics as well as how they challenge external power structures is a further subject of potential academic work.

In the end, it may also be worth considering how a less evangelical view of sport might also benefit policy advocacy activities. Many actors of the sector are still enthralled with notions of the “power of sport”, which in fact may undermine advocacy activities in a world of restricted resources and competing priorities. Greater self-reflection and involvement in progressive advocacy may help organisations both enhance participant outcomes and improve their self-interested advocacy by underscoring their awareness and contributions to the bigger picture rather than merely parroting notions of the “power of sport”.

Overall, our work shows that SFD organisations engage in advocacy primarily to secure funding and communicate their value, with limited involvement in progressive advocacy due to resource constraints or perceived pressure from vested interests. To strengthen policy advocacy, tailored funding, capacity-building initiatives, and coalition-building efforts are needed. At the same time, SFD organisations engaging in advocacy must navigate the delicate balance between funders and policymakers, recognizing that meaningful, sustainable change requires action beyond the pitch itself. To support this, researchers should further explore effective advocacy strategies, coalition dynamics, and areas where SFD advocacy can have the most impact.

## Data Availability

The raw data supporting the conclusions of this article will be made available by the authors, without undue reservation.
