# Corrigendum: Playing for progress: policy advocacy in sport for development

**DOI:** 10.3389/fspor.2025.1619410

**Published:** 2025-05-27

**Authors:** Louis Moustakas, Sarah Carney, Sally-Ann J. Fischer, Alana Richardson, Karen Petry, Arnost Svoboda, Ansley Hofmann, Ben Sanders

**Affiliations:** ^1^ Institute of Sport and Sustainable Development, University of Applied Sciences Kufstein, Kufstein, Austria; ^2^ UNESCO Chair in Inclusive Physical Education, Sport, Fitness and Recreation, Munster Technological University, Tralee, Ireland; ^3^ Institute for European Sport Development and Leisure Studies, German Sport University, Cologne, Germany; ^4^ Department of Social Studies in Kinanthropology, Palacký University Olomouc, Olomouc, Czechia; ^5^ FairPlayPoint, Prague, Czechia; ^6^ The International Platform on Sport and Development, Copenhagen, Denmark

**Keywords:** social impact, power, ideology, funding, sustainable development, politics, advocacy

A Corrigendum on Playing for progress: policy advocacy in sport for development By Moustakas L, Carney S, Fischer S-AJ, Richardson A, Petry K, Svoboda A, Hofmann A, Sanders B (2025). Front. Sports Act. Living 7:1546222. doi: 10.3389/fspor.2025.1546222

**Incorrect Funding**

In the published article, there was an error in the Funding statement. Though the funding source was declared, we omitted a required statement regarding funder responsibility for the findings or opinions in the article. The correct Funding statement appears below.

**Funding**

The author(s) declare financial support was received for the research and/or publication of this article. This study and associated publication were funded by the European Commission via an Erasmus + project grant (101133488). Views and opinions expressed are those of the author(s) only and do not necessarily reflect those of the European Union or the European Education and Culture Executive Agency (EACEA). Neither the European Union nor EACEA can be held responsible for them.

The authors apologize for this error and state that this does not change the scientific conclusions of the article in any way. The original article has been updated.

